# Supplementary material for: Metabolomics by NMR Combined with Machine Learning to Predict Neoadjuvant Chemotherapy Response for Breast Cancer
Source: Cancers (Basel). 2022 Oct 15;14(20):5055. doi: 10.3390/cancers14205055 (PMC9600495; doi:10.3390/cancers14205055)
Supplement: Supplementary file 1 [file cancers-14-05055-s001.zip › cancers-1905018-supplementary.pdf]

Metabolomics by NMR Combined with Machine Learning to Predict Neoadjuvant Chemotherapy Response for Breast Cancer

Marcella R. Cardoso, Alex Ap. Rosini Silva, Maria Cecília R. Talarico, Pedro H. Godoy Sanches, Maurício L. Sforça, Silvana A. Rocco, Luciana M. Rezende, Melissa Quintero, Tassia B. B. C. Costa, Laís R. Viana, Rafael R. Canevarolo, Amanda C. Ferracini, Susana Ramalho, Junier Marrero Gutierrez, Fernando Guimarães, Ljubica Tasic, Alessandra Tata, Luís O. Sarian, Leo L. Cheng, Andreia M. Porcari and Sophie F. M. Derchain

Supplementary Materials

List of Contents:

1. **Supplementary Methods:**

Clinical, histopathologic, and diagnosis of breast cancer.

2. **Supplementary Tables:**

Table S1: Standard NACT regimens and response evaluation parameters according to the molecular subtype of breast cancer.

Table S2: Women's distribution into the therapeutic regimens.

Table S3: Performance for the training and validation sets based on the models built using Logistic Regression and Recursive Feature Elimination considering the different combinations of metabolites, hormone receptor (HR), ki67, and HER2 statuses.

Table S4: Performance for the models built using Logistic Regression and Recursive Feature Elimination considering only triple negative or HER2+ patients.

Table S5: Average relative abundances of the serum metabolites detected by ^{1}H-NMR according to response to NACT.

Table S6: Contribution of the HR, Ki67, HER2, and serum metabolites as predictors of the response to NACT in different models obtained with RFE+LR.

3. **Supplementary Figure**

Figure S1: Pathway Enrichment Analysis for the metabolites found as discriminatory, assuming as relevant those pathways with a *p*-value < 0.05.

1. Supplementary Methods

Clinical, Histopathologic, and Diagnosis of Breast Cancer

The following BC features were evaluated: histological type; grade as per Nottingham classification; ER, PR, Ki67 and HER2 expressions; tumor size classified into T1 (≤ 20 mm), T2 (> 20 mm and ≤ 50 mm), T3 (> 50 mm) and T4 (any size with direct extension to the chest wall and/or skin); lymph node involvement classified as: N0 (no regional lymph node metastasis), N1 (mobile ipsilateral lymph node metastases, axillary levels I, II), N2 (clinically fixed or entangled lymph node metastases I, II, or ipsilateral internal mammary lymph node metastasis) or N3 (axillary level III ipsilateral lymph node metastasis with or without axillary involvement at levels I and II, or metastasis in internal mammary lymph node with level I and II axillary involvement, or supraclavicular lymph node metastasis with or without axillary or internal mammary involvement); distant metastasis by clinical description during outpatient follow-up, taking into consideration the period of diagnosis, being classified as: M0 (without clinical or radiographic evidence of distant metastasis) or M1 (distant metastasis determined clinically and radiographically and/or histologically proven greater than 0.2 mm); clinical stage according to tumor size, axillary involvement and presence of distant metastasis; treatment: drug used; surgery performed on the breast (mastectomy or quadrantectomy) and armpit (axillary dissection or sentinel lymph node biopsy); response to NACT, based on the RCB (Residual Cancer Burden) calculation, in which women with pCR or RCB-I were considered responsive to chemotherapy and women with RCB-II and -III were considered resistant.

2. Supplementary Tables:

Table S1: Standard NACT regimens and response evaluation parameters according to the molecular subtype of breast cancer.

Subtype	Initial Treatment (Neoadjuvant)	Response Evaluation	Additional Treatment (Neoadjuvant)
Luminal A – women post-menopause	hormonal therapy for 2 months	(a) progressive disease<br><br>(b) stable or partial/ complete response	(a) no previous treatment; only surgery<br><br>(b) keep the hormonal therapy for 2 more months before surgery
Luminal A – women pre-menopause or Luminal B HER2–	two cycles of chemotherapy with doxorubicin plus cyclophosphamide	(a) stable or progressive disease<br><br>(b) partial or complete response	(a) paclitaxel for 12 weeks previous surgery<br><br>(b) two cycles of doxorubicin plus cyclophosphamide followed by paclitaxel for 12 weeks previous surgery
Luminal B HER2+ pre- or post-menopause and non-Luminal HER2+	two cycles of chemotherapy with doxorubicin plus cyclophosphamide	(a) stable or progressive disease<br><br>(b) partial or complete response	(a) paclitaxel + trastuzumab for 12 weeks previous surgery<br><br>(b) two cycles of doxorubicin plus cyclophosphamide followed by paclitaxel plus trastuzumab for 12 weeks previous surgery
Triple-negative	two cycles of chemotherapy with doxorubicin plus cyclophosphamide	(a) stable or progressive disease<br><br>(b) partial or complete response	(a) paclitaxel plus carboplatin for 12 weeks previous surgery<br><br>(b) two cycles of doxorubicin plus cyclophosphamide followed by paclitaxel plus carboplatin for 12 weeks previous surgery

Table S2: Women's distribution into the therapeutic regimen.

Treatment Regimen	n	Luminal HER2– (Sens/Resis - % Resis)	Luminal HER2+ (Sens/Resis- % Resis)	Non-Luminal HER2+ (Sens/Resis- % Resis)	Triple Negative (Sens/Resis- % Resis)
Paclitaxel	3	0/1 - 100.0%	0/1 - 100.0%		0/1 - 100.0%
Paclitaxel + Carboplatin	1				0/1 - 100.0%
Paclitaxel + Trastuzumab	1		0/1 - 100.0%		
Cyclophosphamide + Doxorubicin	3	0/3 - 100.0%			
Cyclophosphamide + Doxorubicin + Trastuzumab	1			0/1 - 100.0%	
Cyclophosphamide + Doxorubicin + Paclitaxel	31	1/28 - 96.5%	0/3 - 100.0%		
Cyclophosphamide + Doxorubicin + Paclitaxel + Trastuzumab	26		5/19 - 74.3%	5/7 - 28.6	
Cyclophosphamide + Doxorubicin + Paclitaxel + Carboplatin	14	1/3 - 66.6%		2/2 - 100.0%	4/9 - 55.5%
Total	80	2/35 - 94.3%	5/24 - 79.2%	5/10 - 60%	4/11 - 63.3

Sens: Sensitive
Resis: Resistant

Table S3: Performance for the training and validation sets based on the models built using Logistic Regression (LR) and Recursive Feature Elimination (RFE) considering the different combinations of metabolites, hormone receptor (HR), ki67, and HER2 statuses.

Model #	Features considered	Outcome	Resistant	Sensitive	Sensitivity (%)	Specificity (%)	PPV (%)	NPV (%)	Acc (%)	CI.95
				**Model performance for the training set (48 resistant/12 sensitive)**						
I	Metabolites	Resistant	35	2						
		Sensitive	13	10	73	83	95	43	75	0.62–0.85
II	Metabolites + HR	Resistant	36	2						
		Sensitive	12	10	75	83	95	45	77	0.64–0.87
III	Metabolites + Ki67	Resistant	38	2						
		Sensitive	10	10	79	83	95	50	80	0.68–0.89
IV	Metabolites + HER2	Resistant	40	2						
		Sensitive	8	10	83	83	95	56	83	0.71–0.92
V	Metabolites + HR + Ki67	Resistant	34	2						
		Sensitive	14	10	71	83	94	42	73	0.6–0.84
VI	Metabolites + HR + HER2	Resistant	40	2						
		Sensitive	8	10	83	83	95	56	83	0.71–0.92
VII	Metabolites + Ki67 + HER2	Resistant	40	2						
		Sensitive	8	10	83	83	95	56	83	0.71–0.92
VIII	Metabolites + Ki67 + HER2 + HR	Resistant	37	2						
		Sensitive	11	10	77	83	95	48	78	0.66–0.88
IX	Ki67 + HER2 + HR	Resistant	48	10						
		Sensitive	0	2	100	17	83	100	83	0.71–0.92
				**Model performance for the validation set (16 resistant/4 sensitive)**						
**Model #**	**Predictors**	**Outcome**	**Resistant**	**Sensitive**	**Sensitivity (%)**	**Specificity (%)**	**PPV (%)**	**NPV (%)**	**Acc (%)**	**CI.95**
I	Metabolites	Resistant	14	3						
		Sensitive	2	1	88	25	82	33	75	0.51–0.91
II	Metabolites + HR	Resistant	13	1						
		Sensitive	3	3	81	75	93	50	80	0.56–0.94
III	Metabolites + Ki67	Resistant	15	4						
		Sensitive	1	0	94	0	79	0	75	0.51–0.91
IV	Metabolites + HER2	Resistant	15	3						
		Sensitive	1	1	94	25	83	50	80	0.56–0.94
V	Metabolites + HR + Ki67	Resistant	12	1						
		Sensitive	4	3	75	75	92	43	75	0.51–0.91

VI	Metabolites + HR + HER2	Resistant	16	2						
		Sensitive	0	2	100	50	89	100	90	0.68–0.99
VII	Metabolites + Ki67 + HER2	Resistant	15	3						
		Sensitive	1	1	94	25	83	50	80	0.56–0.94
VIII	Metabolites + Ki67 + HER2 + HR	Resistant	15	2						
		Sensitive	1	2	94	50	88	67	85	0.62–0.97
IX	Ki67 + HER2 + HR	Resistant	16	4						
		Sensitive	0	0	100	0	80	NA	80	0.56–0.94

PPV: Positive Predict Value
NPV: Negative Predict Value
Acc: Accuracy
RFE: Recursive Feature Elimination
LR: Logistic Regression

Table S4: Performance for the models built using Logistic Regression (LR) and Recursive Feature Elimination (RFE) considering only triple negative or HER2+ patients.

		Model Performance (31 resistant/14sensitive)								
**Model #**	**Features considered**	**Outcome**	**Resistant**	**Sensitive**	**Sensitivity (%)**	**Specificity (%)**	**PPV (%)**	**NPV (%)**	**Acc (%)**	**CI.95**
I	Metabolites	Resistant	26	4						
		Sensitive	5	10	84	71	87	67	80	0.65–0.9
II	Metabolites + HR	Resistant	26	5						
		Sensitive	5	9	84	64	84	64	78	0.63–0.89
III	Metabolites + Ki67	Resistant	23	4						
		Sensitive	8	10	74	71	85	56	73	0.58–0.85
IV	Metabolites + HER2	Resistant	23	4						
		Sensitive	8	10	74	71	85	56	73	0.58–0.85
V	Metabolites + HR + Ki67	Resistant	18	2						
		Sensitive	13	12	58	86	90	48	67	0.51–0.8
VI	Metabolites + HR + HER2	Resistant	27	5						
		Sensitive	4	9	87	64	84	69	80	0.65–0.9
VII	Metabolites + Ki67 + HER2	Resistant	23	4						
		Sensitive	8	10	74	71	85	56	73	0.58–0.85
VIII	Metabolites + Ki67 + HER2 + HR	Resistant	31	8						
		Sensitive	0	6	100	43	79	100	82	0.68–0.92

PPV: Positive Predict Value

NPV: Negative Predict Value

Acc: Accuracy

RFE: Recursive Feature Elimination

LR: Logistic Regression

Table S5: Average relative abundances of the serum metabolites detected by ^{1}H-NMR according to response to NACT.

Metabolites	Resistant Mean (Sd)	Sensitive Mean (Sd)	*p*-value*	Fold-Change	Variation
Arginine	38.57 (9.47)	34.03 (8.63)	0.0774	1.13	Up
Aspartate	24.37 (7.07)	21.49 (4.7)	0.0581	1.13	Up
Betaine	18.95 (6.01)	18.03 (7.54)	0.6546	1.05	Up
Carnitine	19.42 (5.45)	19.28 (3.91)	0.9055	1.01	Up
Choline	11.93 (3.12)	11.92 (2.92)	0.9959	1	Down
Citrate	31.28 (8.99)	30.96 (9.27)	0.9003	1.01	Up
Formate	25.4 (7.68)	20.98 (6.38)	**0.0252**	1.21	Up
Glutamate	47.72 (10.82)	43.93 (9.63)	0.1819	1.09	Up
Histidine	28.98 (7.34)	26.71 (7.53)	0.2904	1.08	Up
Isoleucine	31.02 (8.1)	33.28 (9.86)	0.4052	0.93	Down
Pantothenate	8.85 (2.24)	9.65 (3.57)	0.4037	0.92	Down
Phenylalanine	8.44 (3.55)	8.6 (3.98)	0.8903	0.98	Down
Serine	52.78 (12.91)	53.24 (12.29)	0.8947	0.99	Down
Taurine	50.9 (16.49)	48.77 (12.75)	0.578	1.04	Up
Valine	113.59 (22.75)	113.43 (26.24)	0.9825	1	Down
Ascorbate	17.07 (8.5)	15.91 (7.8)	0.6694 (W)	1.07	Up
Asparagine	17.2 (5.03)	16.08 (6.45)	0.576 (W)	1.07	Up
Creatine	19.21 (8.84)	16.55 (7.86)	0.2981 (W)	1.16	Up
Creatinine	18.94 (5.83)	19.92 (6.98)	0.6347 (W)	0.95	Down
Ethanol	52.96 (49.35)	76.4 (108.14)	0.6959 (W)	0.69	Down
Glycerol	60.39 (21.17)	63.43 (25.03)	0.9568 (W)	0.95	Down
Leucine	32.19 (8.16)	37.01 (8.2)	**0.004 (W)**	0.87	Down
Lysine	35.81 (6.89)	35.18 (8.25)	0.9281 (W)	1.02	Up
Proline	126.16 (36.54)	119.63 (35.4)	0.5356 (W)	1.05	Up
Threonine	44.87 (10.81)	41.73 (11.96)	0.2607 (W)	1.08	Up
Tyrosine	22.71 (6.63)	21.25 (6.02)	0.6959 (W)	1.07	Up
myo,Inositol	17.62 (5.4)	18.69 (4.54)	0.339 (W)	0.94	Down
sn,Glycero,3,phosphocholine	22.68 (4.57)	23.92 (4.92)	0.2409 (W)	0.95	Down

p*-values labeled with (W) indicate that Mann–Whitney–Wilcoxon test was used; unmarked *p*-values were calculated using t-tests. SD: standard deviation. FC: fold change. Significant values are in **bold.

Table S6: Contribution of the HR, Ki67, HER2, and serum metabolites as predictors of the response to NACT in different models obtained with RFE+LR.

	Models		Intercept	Leucine	Formate	Proline	Valine	HR-	HR+	Ki67-	Ki67+	HER2-	HER2+
Logit (RT-Res/Sens-)	I	Metabolites	−2.32	2.6	−1.56	−0.65	−2.62	-	-	-	-	-	-
	II	Metabolites + HR	−2.42	2.75	−1.55	−0.8	−2.39	0.5	−0.49	-	-	-	-
	III	Metabolites + Ki67	−2.40	2.74	−1.64	−0.64	−2.76	-	-	−0.13	0.12	-	-
	IV	Metabolites + HER2	−2.49	2.61	−1.42	−0.69	−2.59	-	-	-	-	−0.33	0.34
	V	Metabolites + HR + Ki67	−2.42	2.75	−1.55	−0.81	−2.39	0.5	−0.49	0.02	−0.01	-	-
	VI	Metabolites + HR + HER2	−2.61	2.74	−1.51	−0.84	−2.39	0.5	−0.48	-	-	−0.33	0.33
	VII	Metabolites + Ki67 + HER2	−2.55	2.73	−1.48	−0.69	−2.73	-	-	−0.06	0.06	−0.31	0.33
	IX	Ki67 + HER2 + HR	−1.6	0	0	0	0	0.28	−0.28	0.02	−0.02	−0.30	0.30

RFE: Recursive Feature Elimination

LR: Logistic Regression

3. Supplementary Figures

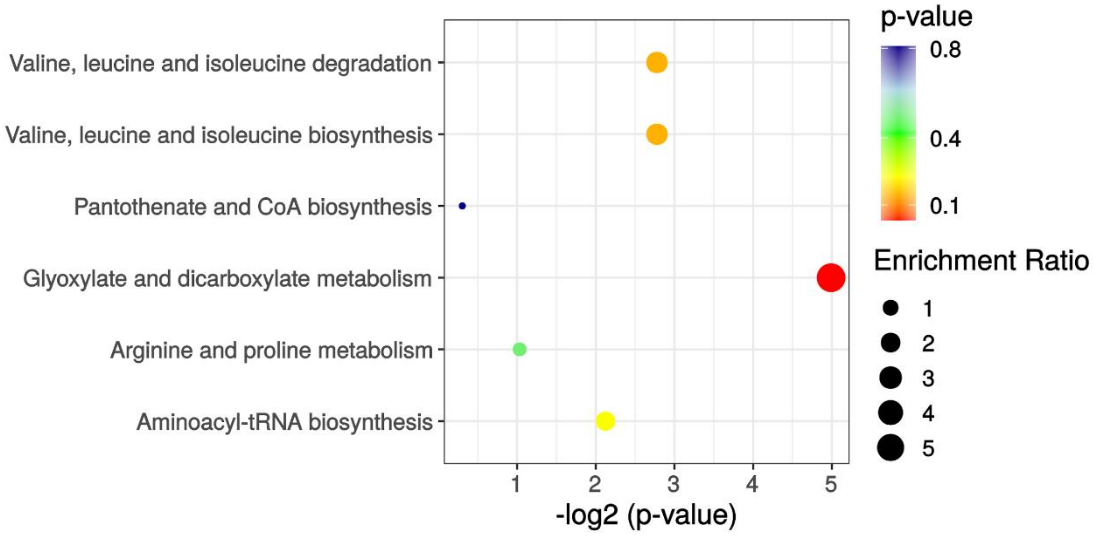

Figure S1. Pathway Enrichment Analysis for the metabolites found as discriminatory, assuming as relevant those pathways with a *p*-value < 0.05.
